# Supplementary material for: Factors associated with patient experiences of the burden of using medicines and health-related quality of life: A cross-sectional study
Source: PLoS One. 2022 Apr 28;17(4):e0267593. doi: 10.1371/journal.pone.0267593 (PMC9049342; doi:10.1371/journal.pone.0267593)
Supplement: Attachment — Submitted filename: Response to Reviewers.pdf [file pone.0267593.s001.pdf]

Reviewers' comments:

---

5. Review Comments to the Author

Please use the space provided to explain your answers to the questions above. You may also include additional comments for the author, including concerns about dual publication, research ethics, or publication ethics. (Please upload your review as an attachment if it exceeds 20,000 characters)

Reviewer #1: The manuscript is written in a meaningful manner systematically. Appropriate statistical analytical tests have been used for obtaining the objectives for which the study was planned for. The authors also have submitted the limitations of their study which are acceptable
On the whole the article is well written and is an evidence towards the outcome, which can be used for a broader study to be utilised for the public practice by the physicians.

Response: The authors would like to thank you for your feedback!

Reviewer #2: 1. Sampling strategy should be mentioned in the methodology.
How the participants were invited?

Response: The following sentence has been added on page 4 (lines 102-104) of the revised manuscript to provide clarification on the sampling strategy utilised for this study.

'A study advertisement was posted on the notice boards located throughout Goulburn Valley Health, and interested participants were asked to contact the study researchers through the contact details provided on the advertisement.'

what could be biased due to non-participation?

Response: The issue of non-participation is commonly seen in cross-sectional study because there is likely to be a group who may be disinclined to participate. Bias generally arises if the survey samples are not representative of the target population. Therefore, the findings of this study are likely to be biased due to non-participation, which could affect the external validity of the study.

The following sentence has been added to page 11 (lines 299-300) of the revised manuscript.

'Thirdly, the bias due to non-participation was likely to affect the external validity of the study.'

2. What is the data collection procedure? Is it self-administered?

Response: Yes, this study involved self-administered questionnaires. Participants were provided with the option to complete the questionnaires available through a paper-based method or an online Qualtrics platform. The same clarifications have been added on page 4 (line 106) of the revised manuscript.

3. The parameters taken for sample size estimation have not been mentioned. Even post-hoc power calculation could be an option.

Response: A short paragraph (also presented below) in relation to Post-Hoc Calculation of Sample Size has been added to page 9 (lines 237-240) of the revised manuscript.

'Post-Hoc Calculation of *Sample Size*

Using G*Power (version 3.1.9.4) post-hoc calculation with a medium effect size, a 5% significance level, six predictors and a sample size of 119 resulting in the power of the study to be 89%.'

4. Kindly check for tense and grammar once.

Response: The authors have done their best in checking for tense and also rectifying grammatical errors.

5. In some references issue number is not mentioned.

Response: The authors have updated the references in the revised manuscript.
